# Successful endoscopic treatment using thulium YAG laser for multiple ureteral fibroepithelial polyps in a pediatric patient

**DOI:** 10.1002/iju5.12432

**Published:** 2022-03-17

**Authors:** Shinji Fukui, Takashi Yoshida, Kazuyoshi Nakao, Tomoaki Matsuzaki, Hidefumi Kinoshita

**Affiliations:** ^1^ 12880 Department of Urology and Andrology Kansai Medical University Hirakata Osaka Japan

**Keywords:** ablation, endoscopic management, multiple fibroepithelial polyps, pediatric, thulium:YAG laser

## Abstract

**Introduction:**

Ureteral fibroepithelial polyps are extremely rare and cause ureteropelvic junction obstruction in the pediatric population. Recent advancements in endoscopic treatment, such as holmium:yttrium–aluminum–garnet laser, have created more options for practitioners to treat multiple ureteral fibroepithelial polyps cases. However, the use of holmium:yttrium–aluminum–garnet laser multilobulated ureteral fibroepithelial polyps may have technical difficulties.

**Case presentation:**

An 11‐year‐old boy with intermittent right flank pain revealed multiple ureteral fibroepithelial polyps approximately 3 cm long at the right ureteropelvic junction. The ureteral fibroepithelial polyps were resected using flexible ureteroscopy using thulium:YAG laser. A second‐look ureteroscopy revealed no recurrence, residual polyps, or ureteral stricture. At 1‐year follow‐up, ultrasonography showed no hydronephrosis on the affected side.

**Conclusion:**

Thulium:YAG laser provides clear visibility due to its high hemostatic and evaporation effects. To the best of our knowledge, this is the first pediatric case of multiple ureteral fibroepithelial polyps successfully treated with endoscopic resection using thulium:YAG laser, with a favorable clinical outcome.

**Abbreviations & Acronyms**
Ho:YAG: holmium:yttrium–aluminum–garnet
Tm:YAG: thulium:YAG
UAS: ureteral access sheath
UFEPs: ureteral fibroepithelial polyps
UPJ: ureteropelvic junction
URS: ureteroscopy

**Keynote message**

Ureteral fibroepithelial polyps (UFEPs) are extremely rare and cause ureteropelvic junction obstruction in the pediatric population. Recent advancements in endoscopic treatment have created more options for practitioners to treat complicated UFEP cases, instead of open or laparoscopic surgery. Thulium:YAG laser provides clear visibility due to its high hemostatic and evaporation effects, leading to complete endoscopic resection of multiple UFEPs.

## Introduction

UFEPs are rare, benign, nonepithelial tumors that originate in the mesoderm of the urinary system. Most UFEPs are located in the distal ureter.^1^, ^2^, ^3^ UFEPs cause ~3.3% of UPJ obstructions and are found in 0.5% of all pediatric patients undergoing pyeloplasty.^3^

Due to complications, such as ureteral stricture that can cause renal failure,^2^ surgical options should be carefully considered for patients with UFEPs. For the pediatric population, open, laparoscopic, or robotic pyeloplasty has been the standard treatment for multiple metachronous UFEPs.^2^, ^3^ With the advancements in endoscopic surgical devices, more reports of endoscopic treatment with Ho:YAG laser have been documented in both adult and pediatric cases with a single UFEP.^2^, ^3^, ^4^ However, using this approach for multilobulated UFEPs may have technical difficulties. Here, we present a case of a child with multiple UFEPs who successfully underwent endoscopic surgery using Tm:YAG laser.

## Case report

**Fig. 1:**
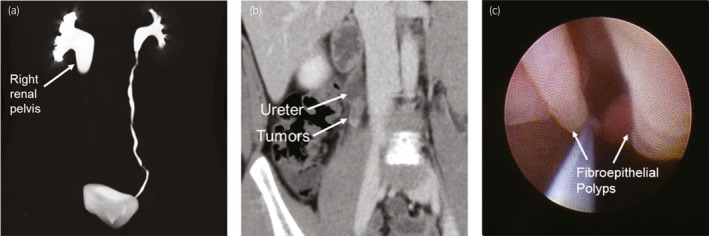
Enhanced computed tomography showing (a) grade 3 hydronephrosis with UPJ obstruction and (b) ureteral tumors at the right UPJ. (c) Diagnostic URS findings of metachronous UFEPs obstructing the right ureter. UFEP, ureteral fibroepithelial polyp; UPJ, ureteropelvic junction; URS, ureteroscopy.

An 11‐year‐old boy presented to our hospital with a complaint of intermittent right flank pain. Computed tomography urography showed grade 3 hydronephrosis with UPJ obstruction (Fig. 1a,b). Diagnostic URS identified three UFEPs of approximately 3.0 cm long at the right UPJ (Fig. 1c). Based on histologic specimens, the UFEPs were pathologically diagnosed as benign tumors. Informed consent regarding treatment options was obtained, and the patient and his family preferred endoscopic laser therapy under general anesthesia because of its less invasive nature.

**Fig. 2:**
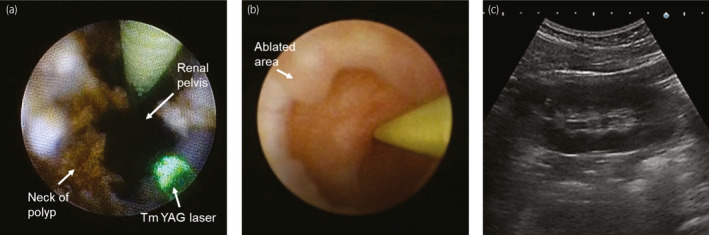
(a) Endoscopic imaging of resection of the UFEP with Tm:YAG laser. (b) Second‐look URS showing no recurrent UFEPs or ureteral stricture 8 weeks after the first URS. (c) Ultrasonography representing no hydronephrosis at 1‐year follow‐up. Tm:YAG, thulium:yttrium aluminum garnet; UFEP, ureteral fibroepithelial polyp; URS, ureteroscopy.

Surgery was performed as follows. A 4.7 Fr ureteral double‐J stent was placed in the patient under general anesthesia 2 weeks before endoscopic surgery in order to facilitate ureteral dilation for insertion of the UAS. During surgery, the tip of a 10/12 Fr UroPass^®^ UAS (Olympus, Tokyo, Japan) was placed distal from the UFEPs using a guidewire and a flexible URF‐P7 ureteroscope (Olympus). Ho:YAG laser (settings: 0.8 J, 10 Hz, long‐pulse mode) was initially used for UFEP resection, but it caused bleeding from the polyps, resulting in poor visibility. Therefore, we used Tm:YAG laser (Revolix120; Lisa laser, Katlenburg‐Lindau, Germany) at 5/15 W to ablate the multiple UFEPs. No bleeding occurred from the ablated polyps, and complete resection was achieved using a basket forceps (Fig. 2a). The operating time was 107 min, and there were no intra‐ or postoperative complications.

Subsequently, 8 weeks later, a second‐look URS was performed, which revealed no recurrence, residual UFEPs, or ureteral stricture (Fig. 2b). At 3‐month follow‐up, ultrasonography showed no hydronephrosis on the kidney’s right side (Fig. 2c). In addition, 1 year after surgery, the patient did not experience ureteral stricture, hydronephrosis, or flank pain.

## Discussion

Currently, there are no formal guidelines on the surgical management of UFEPs. Treatment options include resection via URS using laser, partial ureterectomy, and dismembered pyeloplasty^3^; the right treatment can be determined on the basis of the size and location of the polyps. Ho:YAG laser has been generally used for the endoscopic management of UFEPs^3^ because it has a low rate of tissue perforation due to its shallow depth of penetration (0.4 mm).^5^ However, its pulsed waves and fiber tip vibrations frequently cause bleeding, leading to decreased visibility, imprecise laser ablation, and the risk of incomplete resection of multiple UFEPs.^5^ Li et al.^3^ proposed an algorithm for selecting surgical options for children with UFEPs, indicating that endoscopic treatment is recommended for single, pedunculated UFEPs but not for multilobulated UFEPs, which require dismembered pyeloplasty.

Over the past decade, Tm:YAG laser has been used to treat benign prostate hyperplasia. The central wavelength of Tm:YAG laser is 1.75–2.22 μm, which closely matches the 1.92 μm wavelength that denotes peak water absorption in tissues, resulting in more efficient and rapid tissue cutting. Furthermore, Tm:YAG laser’s continuous wave and shallow penetration (0.4 mm) can provide maximum hemostatic and coagulation effects and a lower risk of tissue perforation, while more effectively destroying tissues.^6^ Recently, Tm:YAG laser was used for endoscopic laser treatment of upper urinary tract carcinoma, and its use is expected to expand to nephron‐sparing surgeries.^5^, ^7^

During laser ablation, small UFEPs (≤5 mm) can be ablated at their pedicles only with Tm:YAG laser. However, for larger UFEPs (>5 mm), such as in this case report, entirely coagulating them using Tm:YAG laser and then resecting them using Ho:YAG (settings: 1 J, 10 Hz, short‐pulse mode) for rapid excision are recommended. To avoid postoperative ureteral stricture, Tm:YAG laser ablation should be performed at low power (5 W) and with a short ablation time near the ureteral mucosa. In addition, instead of vertical ablation, tangent ablation is preferred to prevent direct energy conduction to the ureteral wall.

Some studies have described URS for UFEPs using Tm:YAG laser in adult patients. Sheng et al.^8^ compared the treatment outcomes of adult patients with UFEPs between Ho:YAG laser (*n* = 12) and Tm:YAG laser (*n* = 13) use. The authors reported that three (25%) patients treated with Ho:YAG laser ablation showed ureteral wall perforation and four (33%) developed ureteral stenosis, whereas no patients treated with Tm:YAG laser ablation experienced intra‐ and postoperative severe complications during the 3‐year follow‐up. Gu et al.^9^ reported the multicenter outcomes of 21 adult patients with UFEPs treated with endoscopic Tm:YAG laser ablation. They found that no patient exhibited ureteral perforation intraoperatively and ureteral stricture postoperatively, indicating that Tm:YAG laser ablation is feasible and effective as a minimally invasive surgical method for UFEPs in adult patients.

As shown in our case, using Ho:YAG laser from the beginning to make a UFEP vaporization incision led to bleeding from the UFEPs, causing poor visibility. Compared to Ho:YAG laser, Tm:YAG laser provides maximum hemostatic effects, leading to clear visibility during ablation of multiple UFEPs. Tm:YAG laser also provides a lower risk of iatrogenic ureteral stricture due to its shallow penetration depth.^6^ In our case, the change from Ho:YAG to Tm:YAG laser enabled the resection of multiple UFEPs, maintaining clear visualization during ablation. To the best of our knowledge, this is the first report on URS using Tm:YAG laser for multiple UFEPs in pediatric patients.

## Conclusions

Endoscopic treatment with Tm:YAG laser is likely effective for pediatric patients with multiple UFEPs and is less invasive than conventional open or laparoscopic surgery.

## Author Contributions

Shinji Fukui: Conceptualization; Data curation; Writing – original draft. Takashi Yoshida: Conceptualization; Writing – review & editing. Kazuyoshi Nakao: Conceptualization; Data curation. Tomoaki Matsuzaki: Conceptualization; Investigation. Hidefumi Kinoshita: Conceptualization; Supervision.

## Conflict of interest

The authors declare that they have no conflict of interest.

## Approval of the research protocol

Not applicable.

## Informed consent

Informed consent was obtained.

## Registry and the registration number

Not applicable.
